# A prospective clinical trial of specialist renal nursing in the primary care setting to prevent progression of chronic kidney: a quality improvement report

**DOI:** 10.1186/1471-2296-15-155

**Published:** 2014-09-20

**Authors:** Rachael C Walker, Mark R Marshall, Nick R Polaschek

**Affiliations:** Hawkes Bay District Health Board, Hastings, New Zealand; Sydney School of Public Health, Sydney Medical School, University of Sydney, Sydney, Australia; Counties Manukau District Health Board, Auckland, New Zealand; Faculty of Medical and Health Sciences, University of Auckland, Auckland, New Zealand; Sector Capability and Implementation, Ministry of Health, Wellington, New Zealand

**Keywords:** Chronic kidney disease, Quality improvement, Primary care, Nurse practitioner, Prevention

## Abstract

**Background:**

Early detection and effective management of risk factors can potentially delay progression of chronic kidney disease (CKD) to end-stage kidney disease, and decrease mortality and morbidity from cardiovascular (CV) disease. We evaluated a specialist nurse-led intervention in the primary care setting to address accepted risk factors in a study sample of adults at ‘high risk of CKD progression’, defined as uncontrolled type II diabetes and/or hypertension and a history of poor clinic attendance.

**Methods:**

The study was a non-controlled quality improvement study with pre- and post- intervention comparisons to test feasibility and potential effectiveness. Patients within two primary care practices were screened and recruited to the study. Fifty-two patients were enrolled, with 36 completing 12-months follow-up. The intervention involved a series of sessions led by the nephrology Nurse Practitioner with assistance from practice nurses. These sessions included assessment, education and planned medication and lifestyle changes. The primary outcome measured was proteinuria (ACR), and the secondary outcomes estimated glomerular filtration rate (eGFR) and 5-year absolute CV risk. Several ‘intermediary’ secondary outcomes were also measured including: blood pressure, serum total cholesterol, glycosylated haemoglobin (HbA1c), body mass index (BMI), prevalence of active smoking, a variety of self-management domains, and medication prescription. Analysis of data was performed using linear and logistic regression as appropriate.

**Results:**

There was a significant improvement in ACR (average decrease of −6.75 mg/mmol per month) over the course of the study. There was a small but significant decrease in eGFR and a reduction in 5 year absolute CV risk. Blood pressure, serum total cholesterol, and HbA1c all decreased significantly. Adherence to lifestyle advice improved with a significant reduction in prevalence of active smoking, although there was no significant change in BMI. Self-management significantly improved across all relevant domains.

**Conclusions:**

The results suggest that a collaborative model of care between specialist renal nurses and primary care clinicians may improve the management of risk factors for progression of CKD and CV death. Further larger, controlled studies are warranted to definitively determine the effectiveness and costs of this intervention.

**Trial registration:**

Australian and New Zealand Clinical Trials Registry number: ACTRN12613000791730

**Electronic supplementary material:**

The online version of this article (doi:10.1186/1471-2296-15-155) contains supplementary material, which is available to authorized users.

## Background

Chronic kidney disease (CKD) is a global public health issue. In New Zealand, end stage kidney disease (ESKD) alone accounts for 1-2% of total health care expenditure, a figure comparable to that in the United Kingdom [1, 2]. The group with highest attributable risk of progressive CKD is those with diabetes mellitus, and this risk is exacerbated by hypertension, obesity and dyslipidaemia [3, 4]. In addition, CKD is an independent risk factor for cardiovascular (CV) morbidity and mortality through reduced glomerular filtration rate (GFR) and proteinuria [5, 6]. In New Zealand, indigenous groups (Maori and Pacific peoples) are over represented in their rates of ESKD, accounting for 44% and 29% of those commencing renal replacement therapy in New Zealand, respectively [7]. These indigenous groups also have highest prevalence of CKD and associated risk factors for progression.

It is generally accepted that early detection and effective management of CKD is the prime strategy to reduce progression to ESKD, decrease CV morbidity and mortality, and ultimately limit resource consumption [3, 8–10]. However despite this knowledge, efforts to manage CKD have not proven effective and the number of patients reaching ESKD continues to increase. Within New Zealand, there are numerous reported barriers that hamper effective management of CKD in the community including socio-economic factors, poor accessibility to primary care, poor health literacy, lack of knowledge of CKD and sub-optimally controlled diabetes and blood pressure [11, 12]. Alternative models of healthcare delivery are needed to address these issues.

A promising intervention is adjunctive support from nephrology Nurse Practitioners (NPs), which has been recently shown to reduce the rate of CKD progression and improve renal outcomes [13]. Although this intervention can be regarded as multifaceted, a key element in the role of NPs is coaching to improve self-management by patients. Although the researchers in the aforementioned study did not assess for change in self-management, there have been other randomized control trials (RCTs) involving specifically designed self-management interventions for CKD patients. These have shown measurable improvements in health behaviours and a reduction in the duration and number of hospital admissions [14], although a recent systematic review of literature has highlighted the small effect of self-management interventions upon level of adherence [15].

In 2010 the New Zealand Ministry of Health funded several initiatives to improve outcomes for New Zealanders with CKD through more effective management in the primary care setting. In New Zealand, secondary and tertiary services such as nephrology are provided free of charge to all patients through public hospitals funded by taxation. By contrast, primary care (where most CKD is managed) is provided in private medical practices, where consultations are only partially publicly subsidized. In this article, we describe a quality improvement intervention involving collaboration between a regional nephrology service and local primary care clinicians to manage a group of patients with CKD at high risk of progression. The intervention utilized the resource of a nephrology NP, who in New Zealand is a highly-trained specialist nurse practicing autonomously and able to assess, diagnose and prescribe within their scope of practice [16]. The Ministry of Health funded the time of the NP working with clinicians in their primary care practice, thus enabling the service to be free to the patients.

In this study, we present an evaluation of this intervention in order to demonstrate the clinical potential of the model of care. We employed the reporting framework suggested by the Standards for Quality Improvement Reporting Excellence (SQUIRE) publication guidelines for reporting healthcare quality improvement research (http://squire-statement.org/assets/pdfs/SQUIRE_guidelines_table.pdf) [17].

## Methods

### Ethical issues

The study was approved by the Regional Ethics Committee (IRB00008714) of the New Zealand Ministry of Health (IORG0000895), conditional upon local ethics committee approval which was granted by the Institutional Review Board of the Hawke’s Bay District Health Board after a full review of the study protocol. Patients were invited to participate in the intervention, given verbal and written information regarding the study and what their involvement would include, and informed that they were able to withdraw from participation at any stage.

### Setting

The geographical setting for this study was Hawke’s Bay, New Zealand, a rural district situated on the east coast of New Zealand with a population of approximately 170,000. The population of Hawke’s Bay is notable for a high degree of socioeconomic deprivation, with 26% of the population in the lowest two national deciles of deprivation. The area is also notable for a high prevalence of New Zealand Maori (the indigenous ethnic group), comprising 25% of the population compared to 15% nationally.

The intervention in this study was conducted by Hawke’s Bay District Health Board Nephrology Service in collaboration with two primary care practices located in the highest areas of socioeconomic deprivation in the region. Each practice served a catchment at the time of between 5000–7000 people, with clinicians that included General Practitioners (GPs) and practice nurses, registered nurses employed to work with GPs within the practice.

Patients were studied over a 12 month period, with an accrual period from 1^st^ June 2011 to 15^th^ September 2011.

### Planning the intervention

**Figure 1:**
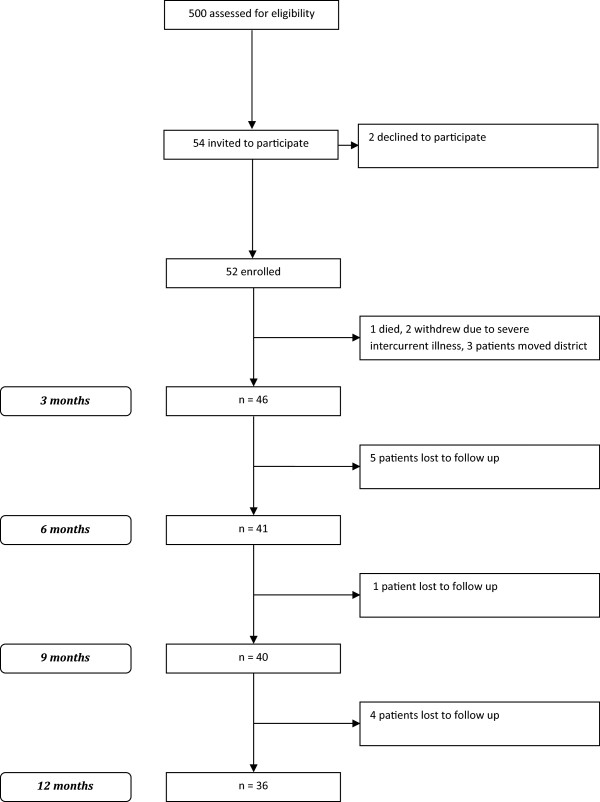
**Patient flow.**

Patients were screened and recruited through the primary care practices. Inclusion criteria required that all of the following conditions were met: ‘high risk of CKD progression’ (as defined below), age >18 years, diagnosis of type two diabetes mellitus, hypertension, and albuminuria defined as an albumin to creatinine ratio (ACR) >30 mg/mmol on at least three occasions separated by at least 1 week [18]. The main exclusion criterion was CKD due to renal parenchymal disease other than diabetic nephropathy. Patients at "high risk of CKD progression" were defined as those with at least 12 months of uncontrolled diabetes and/or hypertension (glycoslyated haemoglobin (HbA1c) >8% and blood pressure (BP) >140/90 mmHg [19, 20]) *and* a history of poor attendance and engagement with their GP (history of unplanned non-attendance of 25% or more of scheduled appointments over the course of 12 months). Over 500 patients were identified from the initial screen within the practices and fifty-four high risk patients were identified by the primary care teams as also meeting the criteria for poor attendance. These fifty-four were given written information and invitation to participate by their GP or practice nurse. All were subsequently re-contacted by phone to answer any questions, and offered an initial assessment. Fifty two patients subsequently enrolled and participated in the study (Figure 1).

The intervention involved a series of sessions led by the nephrology NP with the assistance of the practice nurse. Patients were seen fortnightly for 12 weeks by the NP and the practice nurse, followed by a monitoring phase to 12 months. At baseline, a comprehensive patient history, health literacy and self-management assessment, physical assessment, and laboratory review was performed. Patient history included: medical history, family and social history and lifestyle behaviours including diet, smoking status, salt intake, exercise, and current knowledge of condition and medication. Physical assessment included office measurement of BP according to standardised protocol (JNC 7 [21]), pulse, height, weight, review of home capillary blood sugar records, and clinical cardiac assessment, conducted by the NP. Laboratory review included HbA1c, serum creatinine, estimated glomerular filtration rate (GFR), ACR and serum lipid levels.

Initial sessions involved tailored education and the development of individualised care plans based on best practice guidelines and using self-management and patient-centered theory utilizing the Flinders Chronic Care model [22]. A detailed patient education package was developed for the study and included information on diabetes and its complications, blood pressure management, lifestyle modifications, medication adherence, smoking cessation and dietary advice including low salt intake (dietary sodium intake less than 2.3 g/day). All patients were also given a booklet on self-management developed for the study where they could record all clinical results, self-care goals, individualised medication charts and other important information.

Subsequent sessions involved implementation of the individualized care plans, re-assessment of patients and management plan changes and implementations as required. A stepwise BP protocol was developed for the project with titration of antihypertensives at each fortnightly review to target a BP of 130/80 mmHg [19, 20, 23]. The protocol of medication escalation involved the stepwise addition of angiotensin converting enzyme inhibitors (ACEi) or angiotensin receptor blockers (ARB), thiazide diuretic, calcium channel blocker, and beta blocker or alpha blocker. Patients were provided with free transport to their study appointments, and all medications subjected to usual patient payments and subsidies without additional cost or reimbursement. All patients in the study continued to receive usual health care (appointments as requested by patients or scheduled chronic disease management routine appointments, which are in general less than 15 minutes and 3 monthly at most) from their GP and primary health care team. Patients underwent baseline and three monthly assessments of study end-points over the period of observation. All patient information and results was entered directly into the primary care health care record.

### Planning the study of the intervention

We chose a validated method of measuring and describing the co-morbidities and multi-morbidity in the patient cohort. We extracted co-morbidity data from the medical history of patient records, and classified multi-morbidity using the Cumulative Illness Rating Scale (CIRS) [24]. This scale has been validated and applied in primary care settings, and has been used widely including the Australasian setting [25]. The scale rates the presence and severity of illness in 14 organ systems to provide an index of total chronic medical illness burden [26]. In this study, we assessed multimorbidity using 3 operational definitions: namely, 2 or more diagnoses (or CIRS domains), 3 or more diagnoses (or CIRS domains), and 4 or more diagnoses (or CIRS domains). We assessed the socioeconomic status of the patient cohort using the NZDep score, which combines nine variables from the census that reflect eight domains of deprivation (income, home ownership, social support, employment, academic qualifications, living space, access to a telephone, access to a car). The index provides a score for each meshblock in New Zealand, which are defined geographical areas defined by Statistics New Zealand containing a median number of approximately 87 people in 2006. The NZDep score divides New Zealand into deciles, e.g. a value of 10 indicates the meshblock is in the most deprived 10% of the New Zealand population, and a value of 1 indicates that the meshblock is in the least deprived [27].

We assessed the outcomes of implementing the quality improvement program through the attendance of the participants to appointments, the attendance of both the NP and the practice nurse for the combined clinics, and staff satisfaction.

We measured the overall clinical impact of the quality improvement program through assessment of study outcomes over the 12 month period of the intervention. Measurements of outcomes (other than for health knowledge, medication knowledge, and self-management) were made at baseline, and endpoints consisted of measurements at 3, 6, 9 and 12 months. Measurements of health knowledge, medication knowledge, and self-management were made at baseline, and endpoints consisted of measurements at 3 and 12 months.

The primary outcome was proteinuria, assessed as the ACR on a random urine specimen taken prior to or at the combined clinic consultation. The secondary outcomes were: estimated GFR as assessed from serum creatinine and the 175 4-variable MDRD equation [28] (which to date remains the standard method rather than the CKD-EPI formula throughout New Zealand), and 5-year absolute cardiovascular risk (defined as the likelihood of a cardiovascular event over 5 years) [23].

We also assessed ‘intermediary’ secondary outcomes. These outcomes were either related to processes of care, or related to clinical outcomes accepted as being intermediate on casual pathways leading to either progression of CKD or to increased cardiovascular risk. Such outcomes were chosen as being the most likely mechanisms through which the study intervention might improve outcomes.

For intermediary secondary outcomes related to the participant, we assessed BP according to standardised protocol (JNC 7 [21]), serum total cholesterol, BMI, HbA1c and the prevalence of active smoking through patient clinical records. We assessed overall self-management, overall medical knowledge (knowledge of condition and medication), adherence to medication and adoption of a healthy lifestyle through a self-management reporting scale (Partners In Health Score, PIH score) [29]. The PIH score and questionnaire was developed for the Australian healthcare context, and is used to assess changes in patient self-management knowledge, skill and ability. The assessments are made from both the patient’s own perspective and from the perspective of the treating clinician. The ratings span across twelve domains, or areas of patient knowledge and health-related behaviour. The PIH scores provide a validated longitudinal record of how well patients are coping with and managing their chronic conditions [29].

For intermediary secondary outcomes related to the provider, we assessed prescribing patterns through patient clinical records.

Laboratory measurements were made in a central laboratory using standardized equipment (Abbott Aeroset®, Abbott Laboratories (N.Z.) Limited, Auckland, New Zealand). Serum creatinine assays were performed using the Jaffe method, and calibrated to isotope dilution mass spectroscopy.

### Methods evaluation and analyses

The study data are in the form of a cross-sectional time-series, otherwise known as a panel, with repeated clinical observations obtained from the same patient over time. The data produce an unbalanced panel, as a result of missing observations pertaining to both truly missing data and also observations that are recorded during some months but not others. Of note, all study data from all participants were modelled, including baseline and follow-up results for those who died and dropped out, up until the time of their termination within the study.

We conducted the analysis of study data using regression models within Stata 12.1 MP software (College Station, TX, USA). For continuous data, we used linear models (xtreg procedure) and for categorical ones we used logistic models (xtlogit procedure). To account for internal correlation between repeated patient observations, a random effect model was used for each dependent variable that included all data from all time periods simultaneously, with observations over time from the same patient sharing the same random effects, assuming different random effects for different patients. To account for non-response bias, regression coefficients were estimated using the maximum likelihood method. Time was modelled as a continuous variable, and all coefficients are the modelled change in each parameter per month, with the p value referring to the significance of this change over time.

## Results

### Participants

Of the original 52 participants, 36 were still available for follow-up 12 months later (see Figure 1). Baseline clinical characteristics of participants are provided in Table 1, and details of their total chronic medical illness burden in Additional file 1: Table S1. Socio-economic status of participants is described in Table 2, and illustrates the high level of deprivation with 84% of participants living in the 9 or 10^th^ decile.

**Table 1:** **Clinical characteristics of the study cohort at baseline**

Variable		
Number	*N*	52
Practice	A	28 (54)
	B	24 (46)
Age	Years	57.5 (47–64)
Gender	Male	25 (48)
	Female	27 (52)
Ethnicity	New Zealand European	5 (10)
	Cook Island Maori/Samoan	10 (19)
	New Zealand Maori	37 (71)
Appropriate secondary specialist care		15 (29)
Total chronic medical illness burden	CIRS Score	6.92 (2.27)
2 or more diagnoses (CIRS domains)	%	33
3 or more diagnoses (CIRS domains)	%	3
4 or more diagnoses (CIRS domains)	%	0
Albumin to creatinine ratio	mg/mmol	34.9 (14.2-150.9)
5-year absolute cardiovascular risk	%	20 (15–27)
Estimated GFR	mL/min/1.73 m^2^	63 (48–77)
Systolic BP	mmHg	150 (144.5-160)
Diastolic BP	mmHg	90 (80–110)
Serum total cholesterol	mmol/L	5.25 (4.1-6)
Glycosylated haemoglobin	%	8.8 (7.7-10.7)
Body mass index	kg/m^2^	37 (32.5-43.5)
Active smoking		18 (35)
Self-management	Score	
Overall score (scale 0 to 104)		82 (72–91)
Knowledge of medications (scale 0 to 8)		6 (5–8)
Knowledge of condition (scale 0 to 8)		6 (4–7)
Medication adherence (scale 0 to 8)		8 (5–8)
Healthy lifestyle (scale 0 to 8)		6 (4–8)
Prescribed aspirin	%	31 (60)
Prescribed ACEi / ARBs	%	45 (87)
Prescribed HMG-CoA reductase inhibitors	%	32 (62)
Number of prescribed anti-hypertensives	*n*	2 (1–3)

All data are presented as n (%) or median (IQR).

**Table 2:** **Frequency of New Zealand deprivation scores**

Deprivation score		Frequency	Percent	Cumulative percent
	1	1	1.9	2.0
	2	1	1.9	4.0
	3	1	1.9	6.0
	4	1	1.9	8.0
	7	1	1.9	10.0
	8	3	5.8	16.0
	9	13	25.0	42.0
	10	29	55.8	100.0
	Total	50	96.2	
Missing		2	3.8	
Total		52	100.0	

### Course and success in implementation of intervention

Attendance of both the NP and the practice nurse was required as an integral component of the study intervention, and was 100% for the combined clinics. Staff satisfaction was surveyed as a routine part of clinical operations at the primary care practices, and was reported as high for all practice nurses and GPs involved in this initiative. All patients who were lost to follow up either died, withdrew due to severe intercurrent illness (one stroke, one myocardial infarction), or moved out of the district. ‘Did not attend’ rates were less than 5% for all participants remaining eligible over the entire period of observation.

### Outcomes

Results of regression modelling and rank sum testing for study outcomes are provided in detail in Table 3.

**Table 3:** **Change in observed outcome as a function of time**

Primary outcome	Change per unit per month (linear), or change in odds ratio per month (binary categories)	(95% Confidence Interval)	P Value
ACR	−6.75	−10.98, −2.52	0.002
**Secondary outcomes**			
Estimated GFR	−0.34	−0.55, −0.12	0.002
5-year absolute cardiovascular risk	−0.24	−0.40, −0.09	0.002
**“Intermediary” secondary outcomes - participant**			
Systolic blood pressure (mmHg)	−1.65	−2.02, −1.28	<0.01
Diastolic blood pressure (mmHg)	−1.07	−1.33, −0.81	<0.001
Serum total cholesterol (mmol/L)	−0.05	−0.08, −0.02	0.002
HbA1c (%)	−0.09	−0.13, −0.06	<0.001
BMI (kg/m^2^)	−0.06	-.13, −0.008	0.08
Active smoking (odds ratio)	0.69	0.54, 0.88	0.003
Self-management			
Overall score (scale 0 to 104)	1.11	0.72, 1.50	<0.001
Knowledge of medications (scale 0 to 8)	0.17	0.12, 0.22	<0.001
Knowledge of condition (scale 0 to 8)	0.14	0.10, 0.18	<0.001
Medication adherence (scale 0 to 8)	0.05	0.001, 0.09	0.044
Healthy lifestyle (scale 0 to 8)	0.06	0.02, 0.11	0.005
**“Intermediary” secondary outcomes - provider**			
Prescribed aspirin (odds ratio)	1.61	1.23, 2.11	<0.001
Prescribed ACEi/ARB (odds ratio)	1.97	1.04, 3.72	0.037
Prescribed HMG-CoA reductase inhibitors (odds ratio)	1.26	1.08, 1.46	0.003
Number of prescribed anti-hypertensives	0.05	0.03, 0.07	<0.001

**Figure 2:**
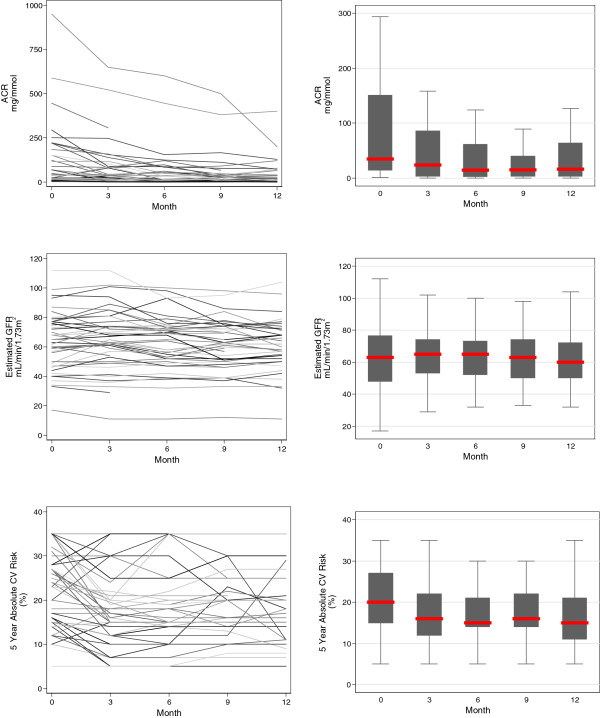
**Primary and secondary outcomes: albumin to creatinine ratio (mg/mmol), estimated GFR (mL/min/1.73 m** ^**2**^ **) and 5 year absolute cardiovascular risk (%) over the period of observation.** Individual participant trajectories are illustrated in the overlaid line plots in the left panels, and the trajectory for the cohort in the boxplots in the right panels (the central line represents the median, the box the first and third quartile, and the whiskers 1.5 × the interquartile range).

There was a significant change in the primary outcome, ACR, over the course of the study, amounting to an average decrease of −6.75 mg/mmol per month over the period of observation. There were also significant changes in the secondary outcomes. There was a significance decrease in estimated GFR of −0.3 mL/min/1.73 m^2^ per month, indicating that progression of CKD was still occurring within the study cohort amounting to a loss of 3–4 mL/min/1.73 m^2^ per annum. There was a significant decrease in the 5 year absolute cardiovascular risk by −0.2% per month. Changes in these primary and secondary outcomes are illustrated in Figure 2.

**Figure 3:**
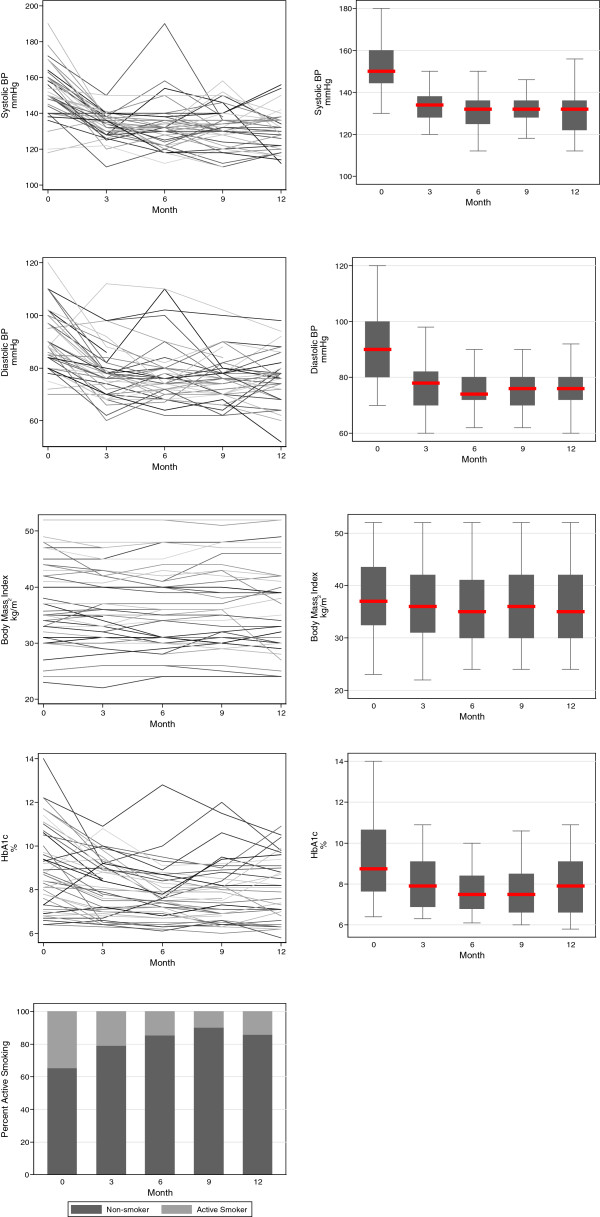
**‘Intermediary’ secondary outcomes related to the patient: BP (mmHg), serum total cholesterol, BMI (kg/m** ^**2**^ **), HbA1c (%), prevalence of active smoking over the period of observation.** For continuous variables, individual participant trajectories are illustrated in the overlaid line plots in the left panels, and the trajectory for the cohort in the boxplots in the right panels (the central line represents the median, the box the first and third quartile, and the whiskers 1.5 × the interquartile range). For categorical variables, bar plots indicate proportions for the entire cohort.

**Figure 4:**
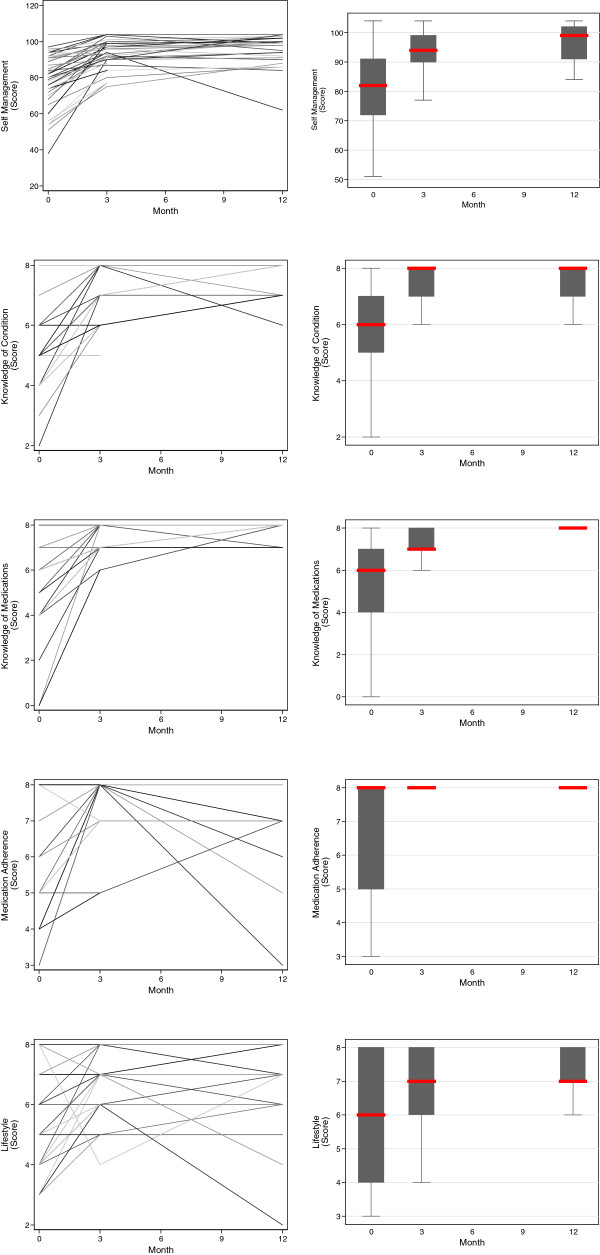
**‘Intermediary’ secondary outcomes related to the patient: self-management, medical knowledge (knowledge of condition and medication), adherence to medication, and adoption of a healthy lifestyle over the period of observation.** Individual participant trajectories are illustrated in the overlaid line plots in the left panels, and the trajectory for the cohort in the boxplots in the right panels (the central line represents the median, the box the first and third quartile, and the whiskers 1.5 × the interquartile range).

**Figure 5:**
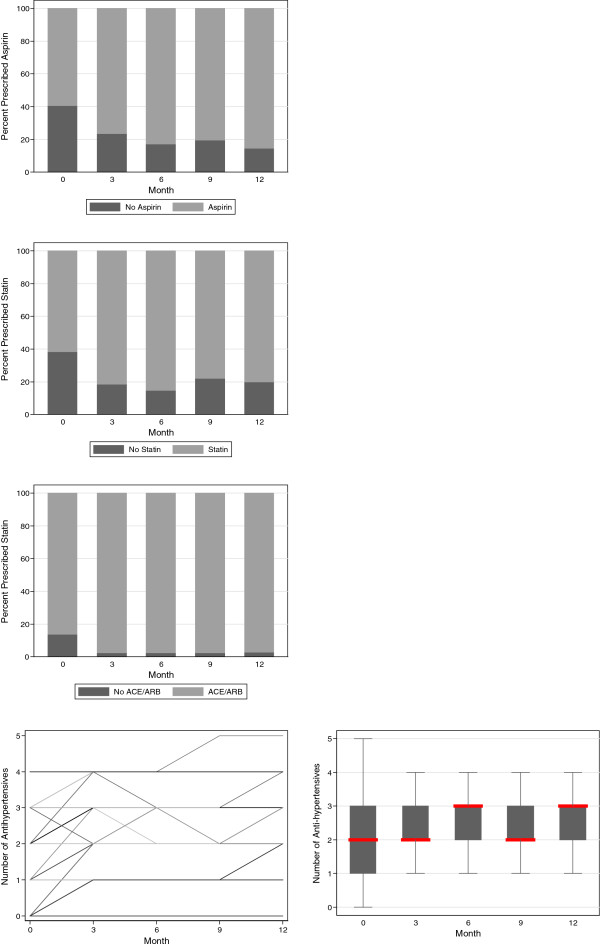
**‘Intermediary’ secondary outcomes related to the provider: prescription of angiotensin converting enzyme inhibitors (ACEi) or angiotensin receptor blockers (ARB), aspirin and lipid lowering medications, and number of antihypertensive medications.** For continuous variables, individual participant trajectories are illustrated in the overlaid line plots in the left panels, and the trajectory for the cohort in the boxplots in the right panels (the central line represents the median, the box the first and third quartile, and the whiskers 1.5 × the interquartile range). For categorical variables, bar plots indicate proportions for the entire cohort.

There were significant changes in intermediary secondary outcomes related to the participant. Blood pressure decreased significantly over the course of the study, with a median baseline measurement of 150/90 and a corresponding 12 month measurement of 132/76. Serum total cholesterol and HbA1c also decreased significantly, with median baseline measurements of 5.25 mmol/L and 8.75%, respectively, and corresponding 12 month measurements of 4.6 mmol/L and 7.55%, respectively. Adherence to lifestyle advice improved, with a significant decrease of active smoking from 35% to 10%, although there was no significant change in BMI. Self-management significantly improved across all relevant domains, with an increase in median overall self-management score from 82 to 99 over the period of observation. These changes are illustrated in Figures 3, 4 and 5.

There were significant changes in intermediary secondary outcomes related to the provider. The prescription of ACEi/ARBs, aspirin and lipid lowering medications, increased significantly to 97%, 86%, and 80% respectively. The mean number of prescribed anti-hypertensives increased significantly from 2 to 3.

## Discussion

This report evaluates a primary care based intervention for CKD patients at high risk of CKD progression. The intervention utilized the nephrology NP within this setting to improve risk factors for progression of CKD. The baseline data for this group of participants indicated sub-optimal management for an extended period of time, with clinical care and outcomes that were not meeting customary targets suggested in clinical practice guidelines. Such characteristics define a common, problematic group of patients, both within New Zealand and internationally. One study from the United Kingdom typifies this experience, with only a fifth of those with diabetes and CKD having a BP of 130/80 mmHg or less, and fewer than half on angiotensin-converting-enzyme inhibitor or angiotensin-receptor blocker [30]. Similar findings have been reported from the United States [31].

This study supports the feasibility and potential effectiveness of an integrated model of care for management of CKD. During the course of the nurse-led intervention there was improved control of accepted risk factors for progression of CKD. Patient adherence to medication and lifestyle advice both improved over the duration of the intervention, indicating that high risk patients are sometimes willing to engage in lifestyle modifications when afforded adequate support and education about management of their condition, combined with empowerment through improved self-management skills.

Several other studies have shown similar success. An algorithm-based, primary care disease-management programme for patients with CKD in the United Kingdom also associated with better control of BP, lower serum cholesterol, and reduced the rate of kidney function loss supporting the notion of a community based model of care as being both efficacious and cost-effective [32]. A community based (but not primary care based) initiative within New Zealand has also reported similar results in this population, with improvements in both renal and cardiac endpoints [33]. A RCT involving intensified NP involvement reduced the decline in kidney function and improved renal outcomes in patients with prevalent CKD over a sustained follow-up period [13].

The intervention described in this study involved collaboration between the regional secondary nephrology service and primary care practices. The nephrology NP worked primarily with practice nurses in the GP practices, with liaison with GPs as required. Interactions between these clinicians allowed for education around best practice and evidence-based management of CKD, and provided opportunities for learning through case reviews. This collaborative model also had the effect of enhancing the currently limited linkages between these primary care practices and the secondary nephrology service in the region.

There is no study in the literature that can be used to determine effect size between improved self-management and improved clinical outcomes. Other studies have assessed self-management as an outcome in itself, but only two have assessed effect on patient-centred outcomes such as HRQoL [34, 35]. Ours is the first to have assessed effect on clinical outcomes. One could question the clinical significance of the small improvement in self-management that we observed in this study, although we note that as with other studies that there is sustained improvement of clinical markers over time, suggesting a sustained and long-term effect that might persist and even increase beyond the period of observation in this study [13]. The improvement in clinical outcomes observed in this study are small but significant. For instance, although the decrease in the 5 year absolute cardiovascular risk seems small at −0.2% per month, this is equivalent to a 1.2% annual decrease in the risk of a cardiovascular event for this study sample. This effect should be considered in the light of the global burden of diabetic patients with CKD, which is the primary diagnosis causing kidney disease in 20–40% of people starting treatment for ESKD [36]. The rates of progression of newly diagnosed type 2 diabetics between the stages of normoalbuminuria, microalbuminuria, macroalbuminuria and kidney failure are 2–3% per year, as evidences in the United Kingdom Prospective Diabetes Study [37]. The annual mortality rate in this population is approximately 7% [38], and at least 50% of these deaths will be cardiovascular in nature. As such, a seemingly small 1-2% annual decrease in the risk of cardiovascular morbidity and mortality has the potential to avert many life-years lost, and many health-dollars spent.

The total cost of the pilot was $160,000 over two years, this included; NP and practice nurse release time, payment for GP’s and nurses to attend regular planning meetings, patient transport, blood tests, administration costs, clinical and electronic equipment and support, printing and development of resources, administration and reporting time. A large proportion of the cost was used in planning the pilot in the six months prior. Further studies should provide more detailed cost benefit analysis.

The success of the intervention suggests a goal for secondary nephrology services, in using their expertise to up-skill primary care clinicians to better manage CKD. There are several ways in which primary care practices can limit the growth of ESKD. Most importantly, practices can better screen their patient populations to identify those at different levels of risk, and work to apply evidence-based medicine to improve risk factor management amongst those with CKD. There are several major financial barriers to the scaling and implementation of programs such as the intervention in this study. The main ones relate to the impact on primary health care clinicians’ work, and the corresponding impact for nephrology NPs who spend time away from their customary duties in secondary care. Self-management education should be included in all aspects of primary care and CKD management as they have proven successful in improving adherence outcomes in other chronic conditions [39, 40].

As a pilot intervention, this nurse-led intervention has potential to limit growth of expensive renal replacement therapy programmes. However, it was supported on a time-limited basis by the New Zealand Ministry of Health, with funding primarily to allow deployment of a clinical resource, the nephrology NP, from secondary into primary care. The service was therefore free to the patients enrolled in the intervention, which we assume contributed to their compliance with the requirements of the programme. The feasibility of reproducing this pilot on a larger scale with an economic benefit would need to be further explored in future research. Making publicly subsidized funding of primary care contingent on clinical outcomes for practice populations may be required to incentivize adoption of different clinical activities. Financial incentive schemes employed in the United Kingdom have been shown to potentially contribute to the reduction in health inequalities in deprived areas [41].

There are several limitations of study design. Firstly, this study is a quality improvement initiative rather than a randomized controlled trial, with no comparator group or clinical data from the period prior to the intervention. As such, there is risk of bias and confounding, and no absolute certainty that the improvements were the direct results of the intervention. It is possible that they arose due to a separate and unrecognized co-intervention. It is also plausible that the improvements arose from “regression to the mean” in our study sample, which can occur when subjects are selected with outcome measures at the extremes of a given distribution. In this situation, the measures will tend to be closer to the centre of the distribution on subsequent measurements, which can often be incorrectly inferred as being an improvement in response to an intervention. The sampling frame in this study (non-adherence over the prior one-year period) reduces the risk of this occurrence, although it remains a very definite, unquantifiable limitation on the interpretation of our results. The second limitation of the study design is that it is a small-scale project, and cannot therefore be considered as proof of clinical effectiveness and cost effectiveness. Instead, the study demonstrates the feasibility of this approach and potential effectiveness. Finally, the study design did not include data collection to evaluate change in the organizational or clinical culture in primary care clinicians outside of the intervention. As such, it is not possible to evaluate whether the intervention was effective in institutionalizing a change in culture towards quality improvement in CKD care [42].

There are several limitations of study analyses that should also be acknowledged. Firstly, these were not performed in a way that allowed for causal inference, which would be the required approach to answer questions such as “did changes in proteinuria result from greater prescription of medication, or rather the improved compliance of said medication?”. Such analyses may be possible using various structural modelling approaches, and will be considered further in the future. Secondly, analyses assume linear relationships over time, a prosaic approach to improve comprehensibility of the statistical models. The analyses cannot, however, address any non-linear relationships that might have occurred over the period of observation. For instance, the changes in many study variables show a pronounced early improvement, with some “rebound” at a later time. Notwithstanding, the models in this study do provide an indication of the overall effect of the intervention over the course of the study, and strongly suggest that the benefits observed over 3 months were sustained to a significant degree during the remainder of the 12 months follow-up. Future studies are planned to identify co-morbidities within the source population of our study sample, to compare multi-morbidity between the groups.

## Conclusions

This study demonstrates that a model of specialist nephrology NP led clinics with primary care clinicians is feasible and may improve risk factors for progression of CKD and cardiovascular death. The cost of implementing such as program a wider basis would be considerable, although costs maybe offset in the long-term if the future burden of ESKD is reduced. Notwithstanding, a collaborative approach to primary and secondary care may be an effective way to manage high risk patients with CKD in the primary care setting. The results of this study call for definitive studies to definitively determine the effectiveness and costs of this intervention in a controlled study on a wider scale.

## Electronic supplementary material

Additional file 1: Table S1: Total chronic medical illness burden of participants, as defined by the Cumulative Index Rating Scale (CIRS) score. (DOC 182 KB)

## References

[1] Ashton T, Marshall MR (2007). The organization and financing of dialysis and kidney transplantation services in New Zealand. Int J Health Care Finance Econ.

[2] Kerr M, Bray B, Medcalf J, O’Donoghue DJ, Matthews B (2012). Estimating the financial cost of chronic kidney disease to the NHS in England. Nephrol Dial Transplant.

[3] Wheeler DC, Becker GJ (2013). Summary of KDIGO guideline: what do we really know about management of blood pressure in patients with chronic kidney disease?. Kidney Int.

[4] Hahr AJ, Molitch ME (2010). Diabetes, cardiovascular risk and nephropathy. Cardiol Clin.

[5] Gerstein HC, Mann JF, Yi Q, Zinman B, Dinneen SF, Hoogwerf B, Hallé JP, Young J, Rashkow A, Joyce C (2001). Albuminuria and risk of cardiovascular events, death, and heart failure in diabetic and nondiabetic individuals. JAMA.

[6] Go AS, Chertow GM, Fan D, McCulloch CE, Hsu C-y (2004). Chronic kidney disease and the risks of death, cardiovascular events, and hospitalization. N Engl J Med.

[7] Grace B, Hurst K, McDonald SP, Hurst K, McDonald SP (2012). Stock and flow. ANZDATA Registry Report 2010.

[8] Black C, Sharma P, Scotland G, McCullough K, McGurn D, Robertson L, Fluck N, MacLeod A, McNamee P, Prescott G, Smith C (2010). Early referral strategies for management of people with markers of renal disease: a systematic review of the evidence of clinical effectiveness, cost-effectiveness and economic analysis. Health Technol Assess.

[9] Collins AJ, Vassalotti JA, Wang C, Li S, Gilbertson DT, Liu J, Foley RN, Chen SC, Arneson TJ (2009). Who should be targeted for CKD screening? Impact of diabetes, hypertension, and cardiovascular disease. Am J Kidney Dis.

[10] Crowe E, Halpin D, Stevens P (2008). Guidelines: early identification and management of chronic kidney disease: summary of NICE guidance. BMJ.

[11] Robinson T, Simmons D, Scott D, Howard E, Pickering K, Cutfield R, Baker J, Patel A, Wellingham J, Morton S (2006). Ethnic differences in Type 2 diabetes care and outcomes in Auckland: a multiethnic community in New Zealand. N Z Med J.

[12] Tomlin A, Tilyard M, Dawson A, Dovey S (2006). Health status of New Zealand European, Maori, and Pacific patients with diabetes at 242 New Zealand general practices. N Z Med J.

[13] Peeters MJ, van Zuilen AD, van den Brand JA, Bots ML, van Buren M, Ten Dam MA, Kaasjager KA, Ligtenberg G, Sijpkens YW, Sluiter HE, van de Ven PJ, Vervoort G, Vleming LJ, Blankestijn PJ, Wetzels JF (2014). Nurse practitioner care improves renal outcome in patients with CKD. J Am Soc Nephrol.

[14] Chen SH, Tsai YF, Sun CY, Wu IW, Lee CC, Wu MS (2011). The impact of self-management support on the progression of chronic kidney disease–a prospective randomized controlled trial. Nephrol Dial Transplant.

[15] Bonner A, Havas K, Douglas C, Thepha T, Bennett P, Clark R (2014). Self-Management Programmes in Stages 1–4 Chronic Kidney Disease: A Literature Review. J Ren Care.

[16] Jacobs SH, Boddy JM (2008). The genesis of advanced nursing practice in New Zealand: policy, politics and education. Nurs Prax N Z.

[17] Davidoff F, Batalden P, Stevens D, Ogrinc G, Mooney S (2008). Publication guidelines for quality improvement in health care: evolution of the SQUIRE project. Qual Saf Health Care.

[18] Caring for Australians with Renal I (2004). The CARI guidelines: Urine protein as diagnostic test: testing for proteinuria. Nephrol.

[19] Chadban S, Howell M, Twigg S, Thomas M, Jerums G, Cass A, Campbell D, Nicholls K, Tong A, Mangos G, Stack A, MacIsaac RJ, Girgis S, Colagiuri R, Colagiuri S, Craig J (2010). The CARI guidelines: Prevention and management of chronic kidney disease in type 2 diabetes. Nephrol.

[20] Harris D, Thomas M, Johnson D, Nicholls K, Gillin A, Caring for Australasians with Renal I (2006). The CARI guidelines: Prevention of progression of kidney disease. Nephrol.

[21] Chobanian AV, Bakris GL, Black HR, Cushman WC, Green LA, Izzo JL, Jones DW, Materson BJ, Oparil S, Wright JT, Roccella EJ (2003). The seventh report of the joint national committee on prevention, detection, evaluation, and treatment of high blood pressure: the JNC 7 report. JAMA.

[22] Harris MF, Williams AM, Dennis SM, Zwar NA, Davies GP (2008). Chronic disease self-management: implementation with and within Australian general practice. Med J Aust.

[23] New Zealand Guidelines Group (2009). New Zealand Cardiovascular Guidelines Handbook: A summary resource for primary care practitioners.

[24] Fortin M, Stewart M, Poitras ME, Almirall J, Maddocks H (2012). A systematic review of prevalence studies on multimorbidity: toward a more uniform methodology. Ann Fam Med.

[25] Britt HC, Harrison CM, Miller GC, Knox SA (2008). Prevalence and patterns of multimorbidity in Australia. Med J Aust.

[26] Hudon C, Fortin M, Soubhi H (2007). Abbreviated guidelines for scoring the Cumulative Illness Rating Scale (CIRS) in family practice. J Clin Epidemiol.

[27] Salmond CE, Crampton P (2012). Development of New Zealand’s deprivation index (NZDep) and its uptake as a national policy tool. Can J Public Health.

[28] Levey AS, Coresh J, Greene T, Stevens LA, Zhang Y, Hendriksen S, Kusek JW, Van Lente F (2006). Using standardized serum creatinine values in the modification of diet in renal disease study equation for estimating glomerular filtration rate. Ann Intern Med.

[29] Battersby MW, Ask A, Reece MM, Markwick MJ, Collins JP (2003). The Partners in Health scale: The development and psychometric properties of a generic assessment scale for chronic condition self-management. Aust J Prim Health.

[30] Stevens PE, O’Donoghue DJ, de Lusignan S, Van Vlymen J, Klebe B, Middleton R, Hague N, New J, Farmer CK (2007). Chronic kidney disease management in the United Kingdom: NEOERICA project results. Kidney Int.

[31] Israni A, Korzelius C, Townsend R, Mesler D (2003). Management of chronic kidney disease in an academic primary care clinic. Am J Nephrol.

[32] Richards N, Harris K, Whitfield M, O’Donoghue D, Lewis R, Mansell M, Thomas S, Townend J, Eames M, Marcelli D (2008). Primary care-based disease management of chronic kidney disease (CKD), based on estimated glomerular filtration rate (eGFR) reporting, improves patient outcomes. Nephrol Dial Transplant.

[33] Hotu C, Bagg W, Collins J, Harwood L, Whalley G, Doughty R, Gamble G, Braatvedt G, Investigators D (2010). A community-based model of care improves blood pressure control and delays progression of proteinuria, left ventricular hypertrophy and diastolic dysfunction in Maori and Pacific patients with type 2 diabetes and chronic kidney disease: a randomized controlled trial. Nephrol Dial Transplant.

[34] Campbell KL, Ash S, Bauer JD (2008). The impact of nutrition intervention on quality of life in pre-dialysis chronic kidney disease patients. Clin Nutr.

[35] Yen M, Huang JJ, Teng HL (2008). Education for patients with chronic kidney disease in Taiwan: a prospective repeated measures study. J Clin Nurs.

[36] U.S. Renal Data System (2013). USRDS 2013 Annual Data Report: Atlas of Chronic Kidney Disease and End-Stage Renal Disease in the United States.

[37] Atkins RC, Zimmet P (2010). Diabetic kidney disease: act now or pay later. Nephrol Dial Transplant.

[38] Barkoudah E, Skali H, Uno H, Solomon SD, Pfeffer MA (2012). Mortality rates in trials of subjects with type 2 diabetes. J Am Heart Assoc.

[39] Janson SL, Fahy JV, Covington JK, Paul SM, Gold WM, Boushey HA (2003). Effects of individual self-management education on clinical, biological, and adherence outcomes in asthma. Am J Med.

[40] Lin EH, Katon W, Von Korff M, Rutter C, Simon GE, Oliver M, Ciechanowski P, Ludman EJ, Bush T, Young B (2004). Relationship of depression and diabetes self-care, medication adherence, and preventive care. Diabetes Care.

[41] Muntner P, Judd SE, Krousel-Wood M, McClellan WM, Safford MM (2010). Low medication adherence and hypertension control among adults with CKD: data from the REGARDS (Reasons for Geographic and Racial Differences in Stroke) Study. Am J Kidney Dis.

[42] Hughes RG (2008). Tools and Strategies for Quality Improvement and Patient Safety.

[CR43] The pre-publication history for this paper can be accessed here:http://www.biomedcentral.com/1471-2296/15/155/prepub

