# Supplementary material for: A prospective clinical trial of specialist renal nursing in the primary care setting to prevent progression of chronic kidney: a quality improvement report
Source: BMC Fam Pract. 2014 Sep 20;15:155. doi: 10.1186/1471-2296-15-155 (PMC4263018; doi:10.1186/1471-2296-15-155)
Supplement: Supplementary file 1 — Additional file 1: Table S1: Total chronic medical illness burden of participants, as defined by the Cumulative Index Rating Scale (CIRS) score. (DOC 182 KB) [file 12875_2014_1135_MOESM1_ESM.doc]

**Additional file 1**

**Table S1.** **Total chronic medical illness burden of participants, as defined by the Cumulative Index Rating Scale (CIRS) score**

| **Patient** | **Cardiac** | **Vascular** | **Hematological** | **Respiratory** | **Ophthalmological and ORL** | **Upper gastrointestinal** | **Lower gastrointestinal** | **Hepatic and pancreatic** | **Renal** | **Genitourinary** | **Musculoskeletal and tegumental** | **Neurological** | **Endocrine, metabolic, breast** | **Psychiatric** | **Total CIRS score** | **>** **=** **2** | **>** **=** **3** | **>** **=** **4** |
| --- | --- | --- | --- | --- | --- | --- | --- | --- | --- | --- | --- | --- | --- | --- | --- | --- | --- | --- |
| 1 | 0 | 2 |  |  |  |  |  |  | 1 |  |  |  | 2 |  | 5 | 2 |  |  |
| 2 |  | 2 |  |  |  |  |  |  | 1 |  |  |  | 2 |  | 5 | 2 |  |  |
| 3 | 1 | 2 |  | 1 |  |  |  |  | 2 |  |  |  | 2 |  | 8 | 3 |  |  |
| 4 |  | 3 |  |  | 1 |  |  |  | 1 |  |  |  | 2 |  | 7 | 1 | 1 |  |
| 5 |  | 2 |  |  | 1 |  |  |  | 1 |  |  | 2 | 2 | 1 | 9 | 3 |  |  |
| 6 |  | 2 |  |  |  |  |  |  | 1 |  |  | 1 | 2 |  | 6 | 2 |  |  |
| 7 |  | 2 |  |  |  |  |  |  | 1 |  | 1 |  | 2 |  | 6 | 2 |  |  |
| 8 | 3 | 2 | 1 | 3 | 1 |  |  |  | 1 |  | 2 |  | 2 |  | 15 | 3 | 2 |  |
| 9 | 2 | 3 |  | 2 | 1 |  |  |  | 1 |  |  |  | 2 |  | 11 | 3 | 1 |  |
| 10 | 2 | 2 |  | 2 | 1 |  |  |  | 1 |  |  |  | 2 |  | 10 | 4 |  |  |
| 11 |  | 2 |  | 3 |  |  |  |  | 1 |  |  |  | 3 |  | 9 | 1 | 2 |  |
| 12 |  | 2 |  |  | 1 |  |  |  | 1 |  |  |  | 2 |  | 6 | 2 |  |  |
| 13 |  |  |  |  |  |  |  |  | 1 |  | 1 |  | 2 |  | 4 | 1 |  |  |
| 14 |  | 2 |  | 2 | 1 |  |  |  | 1 | 1 | 1 | 1 | 2 |  | 11 | 3 |  |  |
| 15 | 2 | 2 |  | 2 | 1 |  |  |  | 1 |  | 1 |  | 2 |  | 11 | 4 |  |  |
| 16 |  | 2 |  |  |  |  |  |  | 1 |  |  |  | 3 |  | 6 | 1 | 1 |  |
| 17 | 2 | 2 |  | 2 |  |  |  |  | 1 |  |  | 2 | 2 | 1 | 12 | 5 |  |  |
| 18 |  | 2 |  |  |  |  |  |  | 1 |  |  |  | 2 |  | 5 | 2 |  |  |
| 19 |  | 2 |  | 3 |  |  |  |  | 1 |  |  |  | 2 |  | 8 | 2 | 1 |  |
| 20 |  | 2 |  |  |  |  |  |  | 1 |  |  |  | 2 |  | 5 | 2 |  |  |
| 21 |  | 2 |  |  |  |  |  |  | 1 |  |  |  | 2 |  | 5 | 2 |  |  |
| 22 |  | 2 |  |  |  |  |  |  | 1 |  |  |  | 2 |  | 5 | 2 |  |  |
| 23 | 1 | 2 |  | 2 |  |  |  |  | 1 |  |  |  | 2 |  | 8 | 3 |  |  |
| 24 | 1 | 2 |  | 2 |  |  |  |  | 1 |  |  |  | 2 |  | 8 | 2 |  |  |
| 25 |  | 2 |  |  |  |  |  |  | 1 |  |  |  | 2 |  | 5 | 2 |  |  |
| 26 |  | 2 |  |  |  |  |  |  | 1 |  |  |  | 2 |  | 5 | 2 |  |  |
| 27 |  | 2 |  | 2 |  |  |  |  | 1 |  |  |  | 2 |  | 7 | 3 |  |  |
| 28 | 2 | 2 |  |  |  |  |  |  | 1 |  |  |  | 2 |  | 7 | 3 |  |  |
| 29 |  | 2 |  |  |  |  |  |  | 1 |  |  |  | 2 |  | 5 | 2 |  |  |
| 30 | 2 | 2 | 1 |  |  |  |  |  | 1 |  | 1 |  | 3 |  | 10 | 2 | 1 |  |
| 31 |  | 2 |  |  | 1 |  |  |  | 1 |  |  |  | 2 |  | 6 | 2 |  |  |
| 32 |  | 2 |  |  |  |  |  |  | 1 |  | 1 |  | 2 |  | 6 | 2 |  |  |
| 33 |  | 2 |  |  |  |  |  | 1 | 1 |  | 1 |  | 2 |  | 7 | 2 |  |  |
| 34 |  | 1 |  | 2 |  |  |  |  | 1 |  |  |  | 2 |  | 6 | 2 |  |  |
| 35 | 2 | 2 |  |  |  |  |  |  | 1 |  | 1 |  | 2 |  | 8 | 3 |  |  |
| 36 |  | 2 |  |  |  |  |  |  | 1 |  |  |  | 2 |  | 5 | 2 |  |  |
| 37 |  | 2 |  |  |  |  |  |  | 1 |  |  |  | 2 |  | 5 | 2 |  |  |
| 38 |  | 2 |  |  |  |  |  |  | 1 |  |  |  | 2 |  | 5 | 2 |  |  |
| 39 |  | 2 |  |  |  |  |  |  | 1 |  |  |  | 3 |  | 6 | 1 | 1 |  |
| 40 |  | 2 |  |  |  |  |  |  | 1 |  | 1 |  | 2 | 1 | 7 | 2 |  |  |
| 41 |  | 2 |  | 3 |  |  |  |  | 1 |  |  |  | 2 |  | 8 | 2 | 1 |  |
| 42 |  | 2 |  | 2 |  |  |  |  | 1 |  |  |  | 2 |  | 7 | 3 |  |  |
| 43 |  | 2 |  | 2 |  |  |  | 1 | 1 |  |  |  | 2 |  | 8 | 3 |  |  |
| 44 |  | 2 |  |  |  |  |  |  | 1 |  |  |  | 2 |  | 5 | 2 |  |  |
| 45 | 2 | 2 |  |  |  |  |  |  | 1 |  |  |  | 2 |  | 7 | 3 |  |  |
| 46 | 1 | 2 |  | 1 |  |  |  |  | 1 |  |  |  | 2 |  | 7 | 2 |  |  |
| 47 |  | 2 |  |  |  |  |  |  | 1 |  |  |  | 2 |  | 5 | 2 |  |  |
| 48 |  | 2 |  |  |  |  |  |  | 1 |  |  |  | 2 |  | 5 | 2 |  |  |
| 49 |  | 1 |  |  |  |  |  |  | 1 |  |  |  | 2 |  | 4 | 1 |  |  |
| 50 |  | 2 |  |  |  |  |  |  | 1 |  |  |  | 2 | 1 | 6 | 2 |  |  |
| 51 |  | 2 |  |  |  |  |  |  | 1 |  |  |  | 2 |  | 5 | 2 |  |  |
| 52 |  | 2 |  | 3 |  |  |  |  | 1 |  |  |  | 2 |  | 8 | 2 | 1 |  |
